# Time-Domain Blind ICI Compensation in Coherent Optical FBMC/OQAM System

**DOI:** 10.3390/s20216397

**Published:** 2020-11-09

**Authors:** Binqi Wu, Jin Lu, Mingyi Gao, Hongliang Ren, Zichun Le, Yali Qin, Shuqin Guo, Weisheng Hu

**Affiliations:** 1 College of Information Engineering, Zhejiang University of Technology, Hangzhou 310023, China; wbq@zjut.edu.cn (B.W.); hlren@zjut.edu.cn (H.R.); qyl@zjut.edu.cn (Y.Q.); guosq@zjut.edu.cn (S.G.); 2 School of Electronic and Information Engineering, Soochow University, Suzhou 215006, China; mygao@suda.edu.cn; 3 College of Science, Zhejiang University of Technology, Hangzhou 310023, China; lzc@zjut.edu.cn; 4 State Key Laboratory of Advanced Optical Communication Systems and Networks, Shanghai Jiao Tong University, Shanghai 200240, China; wshu@sjtu.edu.cn

**Keywords:** coherent optical communication, offset-quadrature amplitude modulation-based filter-bank multicarrier (FBMC/OQAM), blind phase noise compensation, inter-carrier-interference (ICI), discrete cosine transform (DCT)

## Abstract

A blind discrete-cosine-transform-based phase noise compensation (BD-PNC) is proposed to compensate the inter-carrier-interference (ICI) in the coherent optical offset-quadrature amplitude modulation (OQAM)-based filter-bank multicarrier (CO-FBMC/OQAM) transmission system. Since the phase noise sample can be approximated by an expansion of the discrete cosine transform (DCT) in the time-domain, a time-domain compensation model is built for the transmission system. According to the model, phase noise compensation (PNC) depends only on its DCT coefficients. The common phase error (CPE) compensation is firstly performed for the received signal. After that, a pre-decision is made on a part of compensated signals with low decision error probability, and the pre-decision results are used as the estimated values of transmitted signals to calculate the DCT coefficients. Such a partial pre-decision process reduces not only decision error but also the complexity of the BD-PNC method while keeping almost the same performance as in the case of the pre-decision of all compensated signals. Numerical simulations are performed to evaluate the performance of the proposed scheme for a 30 GBaud CO-FBMC/OQAM system. The simulation results show that its bit error rate (BER) performance is improved by more than one order of magnitude through the mitigation of the ICI in comparison with the traditional blind PNC scheme only aiming for CPE compensation.

## 1. Introduction

A coherent optical offset-quadrature amplitude modulation (OQAM)-based filter-bank multicarrier (CO-FBMC/OQAM) system has recently become a promising candidate for high-speed long-haul optical fiber transmission due to its higher spectral efficiency (SE) and its robustness to channel impairments [1,2,3,4,5]. As a multicarrier modulation (MCM) technique, the FBMC is more robust against pulse dispersion and has less out-of-band leakage than orthogonal frequency division multiplexing (OFDM) [6,7,8,9,10,11,12,13,14]. Meanwhile, the FBMC/OQAM scheme does not need cyclic prefix and, therefore, its SE can be increased remarkably [15,16]. 

The performance of optical fiber transmission system can be degraded severely by the phase noise (PN) [17], and the MCM technique is especially vulnerable to PN like OFDM. In CO-FBMC/OQAM systems, the PN generally includes common phase error (CPE) and inter-carrier interference (ICI) [18], similar to that in coherent optical OFDM (CO-OFDM) systems. Nevertheless, the original PN compensation (PNC) algorithms in CO-OFDM cannot be simply transplanted in CO-FBMC/OQAM systems. This is because the OQAM is employed between two immediate adjacent subcarriers, and the orthogonality between subchannels is ensured only in real plane. Due to the nonorthogonality in imaginary plane, the PNC and channel estimation techniques are severely restricted by the so-called intrinsic imaginary interference (IMI) in CO-FBMC/OQAM systems [19,20,21,22,23,24]. Consequently, many studies on the PNC method focus on the IMI cancellation [25,26,27]. In [26], a pilot-based PNC method is used to suppress the IMI at the expense of SE, where a pilot structure consists of several additional data symbols surrounding a pilot symbol. Another alternative approach is a coding scheme, and data symbols surrounding a pilot are deliberately coded to eliminate the IMI in that pilot [26,27]. Although the complexity of two mainly pilot-based PNC methods is moderate, their performance is limited by only CPE compensation.

At present, the PNC methods for CO-FBMC/OQAM systems can be classified into two categories: blind and pilot-aided PNC. As mentioned above, to cancel the IMI, the pilot-aided PNC methods have a low complexity and avoid the effect of cycle slips at the cost of the SE loss [25,26,27]. Compared with the pilot-aided methods, the blind methods can achieve higher SE at the price of higher computational complexity (CC) [28,29,30,31,32,33,34,35,36]. As a de-facto standard in coherent optical communication systems, the blind phase search (BPS) method can achieve a relatively good PNC effect and a high laser linewidth tolerance. Its main disadvantage is high CC resulting from a large number of test phases. The modified BPS (M-BPS) simplifies the distance calculation in the complex plane as the operations in the real plane [28], and thereby reduces the number of multiplications significantly. However, both blind and pilot-aided methods mainly emphasize on the CPE compensation in frequency domain, and the ICI is not considered to be mitigated and is assumed to be additive noise in these studies. Because the MCM system with a large number of subcarriers can increase its fiber chromatic dispersion (CD) tolerance [6], only CPE compensation is obviously not enough in this case due to serious negative impact of the ICI on the system performance [25,26,27,28]. 

Recently, time-domain ICI compensation has been proposed based on pilot symbols in either wireless or optical FBMC/OQAM systems [18,37]. An orthogonal-basis-expansion (OBE)-based PNC method has been proposed in a polarization-division-multiplexed CO-FBMC/OQAM system [18]. With the aid of pilot symbols, the PN in time domain can be mitigated by estimating the corresponding coefficients of the OBE. In wireless FBMC/OQAM systems, by using the discrete cosine transform (DCT) method, the samples of the PN in time-domain can be described as sums of products of a group of DCT bases and their corresponding coefficients of the DCT [37]. Using pilot symbols, the ICI effect can be tracked by calculating these coefficients of the DCT. It is obvious that the pilot overhead loss is very high for two time-domain pilot-based methods. Several blind ICI compensation schemes have been presented for CO-OFDM systems [38,39,40], but they could not be used in CO-FBMC/OQAM systems. 

In this paper, we propose a blind time-domain ICI compensation scheme for CO-FBMC/OQAM systems by combining the M-BPS method and DCT approximation of PN. The M-BPS method is used to obtain the pre-estimated values of the transmitted signals, with which the DCT coefficients can be obtained from the time-domain compensation model. To improve its performance and reduce its CC, the pre-decision error and the overlapped symbol structure are considered to develop the proposed algorithm. The proposed method is numerically validated in a 30 GBaud back to back (BTB) CO-FBMC/OQAM system. The numerical results show that the proposed algorithm achieves a significantly better performance compared to the M-BPS scheme while having a limited increase in CC.

The remainder of this paper is organized as follows. A theoretical model of CO-FBMC/OQAM system with laser phase noise is derived in Section 2. Section 3 introduces the principle of the proposed PNC method. In Section 4, the overlapped symbol structure in the CO-FBMC/OQAM system is implemented to improve the effectiveness of the algorithm. We numerically investigate the performance of our method and propose the complexity analysis in Section 5. Finally, Section 6 summarizes the paper.

## 2. CO-FBMC/OQAM System with Laser Phase Noise

**Figure 1:**
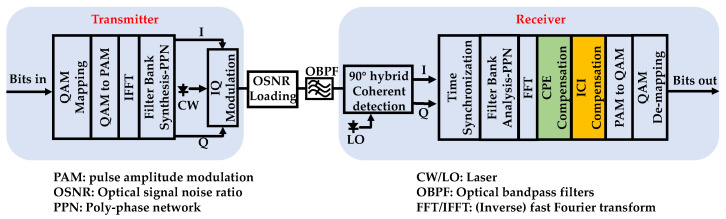
Block diagram of CO-FBMC/OQAM BTB system.

In this section, the influence of PN is studied in CO-FBMC/OQAM systems, and the analytical expressions of the PN induced interference are derived. As shown in Figure 1, a single polarization CO-FBMC/OQAM BTB system is considered to investigate the PN induced interference and the performance of the proposed PNC algorithm. The single polarization BTB system is used to concentrate on the impact of laser PN in this paper.

At the transmitter, the process of OQAM pre-processing transforms the complex-valued QAM symbols into real-valued symbols, which means that the real-valued symbols are formed with the real and imaginary parts of QAM symbols destaggered by half a QAM symbol period. These real-valued symbols are modulated (i.e., pulse amplitude modulation (PAM)) in such a way that the FBMC system maintains the same data rate as the OFDM system without cyclic-prefix. Then, the PAM symbols are mapped onto a set of orthogonal subcarriers generated by an inverse fast Fourier transform (IFFT). The following operation is a cost-efficient poly-phase network (PPN) for filter bank synthesis implementation. With the digital signal processing (DSP) at the transmitter, its discrete time-domain transmitted signal, *s*[*k*], can be expressed as,

(1) $$s[k]=\sum_{n=0}^{N_{S}-1}\sum_{m=0}^{M-1}a_{n,m}g_{n,m}[k]$$

where *a_n,m_* and *g_n,m_*[*k*] are the PAM symbol and the synthesis filter at the (*n*,*m*)th time-frequency index, respectively; *N_S_* is the total number of CO-FBMC/OQAM blocks; and *M* is the total number of subcarriers in each CO-FBMC/OQAM block. The (*n*,*m*)th synthesis filter is described as,

(2) $$g_{n,m}[k]=g[k-n\frac{M}{2}]\cdot e^{j\frac{2\pi }{M}mk}e^{j\psi_{n,m}}$$

(3) $$g[k]=\frac{\left(4\alpha k/T)\cos[\pi (1+\alpha )k/T]+\sin[\pi (1-\alpha )k/T\right]}{\left(\pi k/T)[1-{\left(4\alpha k/T\right)}^{2}\right]}$$

where the square root raised cosine function *g*[*k*] is employed as the prototype filter with length *L_g_* [41], $\psi_{n,m}=(n+m)/2$ is phase modulation factor, α denotes the roll-off coefficient, and T represents the duration of the QAM complex symbols.

In the BTB CO-FBMC/OQAM system, under the impact of laser PN and additive Gaussian white noise (AWGN), the received signal *r*[*k*] can be written as,

(4) $$\begin{array}{cc} r[k] & =e^{j\varphi [k]}s[k]+w[k]\\ & =e^{j\varphi [k]}\left(\sum_{n=0}^{N_{S}-1}\sum_{m=0}^{M-1}a_{n,m}g[k-n\frac{M}{2}]\cdot e^{j\frac{2\pi }{M}mk}e^{j\frac{\pi (m+n)}{2}}\right)+w[k]\\ & =e^{j\varphi [k]}\left(a_{n_{0},m_{0}}g[k-n_{0}\frac{M}{2}]e^{j\frac{2\pi }{M}m_{0}k}e^{j\frac{\pi (m_{0}+n_{0})}{2}}+\sum_{\left(n,m)\neq (n_{0},m_{0}\right)}a_{n,m}g[k-n\frac{M}{2}]e^{j\frac{2\pi }{M}mk}e^{j\frac{\pi (m+n)}{2}}\right)+w[k] \end{array}$$

Here, *φ*[*k*] denotes the PN in the time-domain, and the notation *w*[*k*] is the AWGN. The laser PN originates from either the external cavity laser at the transmitter or the local oscillator at the receiver. It is modeled as a random walk Wiener process, and the PN change follows a Gaussian distribution with zero mean and a specific variance $2\pi \cdot ∆\nu \cdot T_{S}$, where ∆ν is the combined laser linewidth, and *T_S_* is the normalized symbol period. Here, *T_S_* = (1/*B_r_*)/*M*, and *B_r_* is the baud rate of QAM symbols. The combined laser linewidth is defined as the sum of linewidths of the transmitter and receiver lasers. For the sake of simplicity, in the BTB system, the transmitter laser has the same linewidth as the received laser, and the combined laser linewidth is twice the linewidth of the single laser. 

At the receiver side, the incoming optical wave signal is first coherently detected in a 90° optical hybrid. Subsequently, the obtained digital data are sent to the DSP modules to recover the original data. After a time synchronization is performed perfectly, a time-frequency translation can be completed by a hardware-efficient structure of PPN for filter bank analysis followed by a fast Fourier transform (FFT). Eventually, the complex-valued received demodulated signal at the (*n_0_*,*m_0_*)th time-frequency index can be written as,

(5) $$\begin{array}{cc} D_{n_{0},m_{0}} & =\sum_{k=0}^{L_{g}-1}r[k]g_{n_{0},m_{0}}^{*}[k]\\ & =\sum_{k=0}^{L_{g}-1}r[k]g[k-n_{0}\frac{M}{2}]\cdot e^{-j\frac{2\pi }{M}m_{0}k}e^{-j\frac{\pi (m_{0}+n_{0})}{2}} \end{array}$$

here, the upper script “∗” denotes conjugate operation. By substituting Equation (4) into Equation (5), $D_{n_{0},m_{0}}$ can be further expressed as [18],

(6) $$\begin{array}{cc} D_{n_{0},m_{0}} & =\sum_{k=0}^{L_{g}-1}r[k]g[k-n_{0}\frac{M}{2}]\cdot e^{-j\frac{2\pi }{M}m_{0}k}e^{-j\frac{\pi (m_{0}+n_{0})}{2}}\\ & =\eta_{0,0}a_{n_{0},m_{0}}+\sum_{\left(p,q)\neq (0,0\right)}a_{n_{0}+p,m_{0}+q}\eta_{p,q}+N_{n_{0},m_{0}} \end{array}$$

here, $N_{n_{0}}{}_{,m_{0}}$ denotes the filter processed additive noise, and $\eta_{p,q}$ is defined as

(7) $$\begin{array}{ll} \eta_{p,q} & =\sum_{k}e^{-j\frac{\pi (p+q)}{2}}e^{-j\frac{2\pi }{M}qk}g[k-n_{0}\frac{M}{2}]\\ & \cdot g^{*}[k-\frac{M}{2}(n_{0}+p)]\left[\frac{1}{M}\sum_{s=0}^{M-1}e^{-j\frac{2\pi }{M}qs}e^{j\varphi_{n_{0}}[s]}\right] \end{array}$$

at $\left(p,q)=(0,0\right),\;\eta_{0,0}$ is described by the following expression.

(8) $$\begin{array}{ll} \eta_{0,0} & =\sum_{k}g\left[k-n_{0}\frac{M}{2}\right]\cdot g^{*}\left[k-n_{0}\frac{M}{2}\right]\left[\frac{1}{M}\sum_{s=0}^{M-1}e^{j\varphi_{n_{0}}[s]}\right]\\ & =\frac{1}{M}\sum_{s=0}^{M-1}e^{j\varphi_{n_{0}}[s]} \end{array}$$

In Equation (6), $\eta_{0,0}$ is defined as the CPE for the information symbol $a_{n_{0},m_{0}}$, and it leads to phase rotation of the received constellation diagram. The second item of Equation (6) is defined as the ICI, which causes divergence of the received constellation diagram. The ICI in CO-FBMC/OQAM systems is a convolution between the coefficient $\eta_{p,q}$ and PAM symbols from different FBMC/OQAM blocks, while the ICI in CO-OFDM systems is a convolution between the ICI coefficients and different sub-carriers in one OFDM symbol. Hence, it is more difficult to compensate the ICI in CO-FBMC/OQAM systems. 

In many previous studies, assuming that the PN time-domain samples are the same in one FBMC/OQAM symbol period, the complex-valued received signal $D_{n_{0},m_{0}}$ can be approximately rewritten as [25,26,27,28,29],

(9) $$\begin{array}{cc} D_{n_{0},m_{0}} & \approx \eta_{0,0}a_{n_{0},m_{0}}+\eta_{0,0}\sum_{k=0}^{L_{g}-1}\sum_{\left(p,q)\neq (0,0\right)}a_{n_{0}+p,m_{0}+q}g_{n_{0}+p,m_{0}+q}[k]g_{n_{0},m_{0}}^{*}[k]+N_{n_{0},m_{0}}\\ & =\eta_{0,0}(a_{n_{0},m_{0}}+ju_{n_{0},m_{0}})+N_{n_{0},m_{0}} \end{array}$$

where $u_{n_{0},m_{0}}$ is called the IMI in CO-FBMC/OQAM systems, and it is the interference term which results from neighboring PAM symbols on the interested (*n_0_*,*m_0_*)th position. Based on the approximation expression above, various studies have focused on the cancellation of the IMI by using pilot symbols [25,26,27]. However, only CPE compensation effect can be improved through the complete removal of the IMI. Because the ICI effect is the convolution in frequency domain, it is useless to perform the ICI compensation by removing the IMI thoroughly. So in the rest of the paper, the blind ICI compensation is proposed for the CO-FBMC/OQAM systems. 

## 3. Principle of the Phase Noise Compensation Method

**Figure 2:**
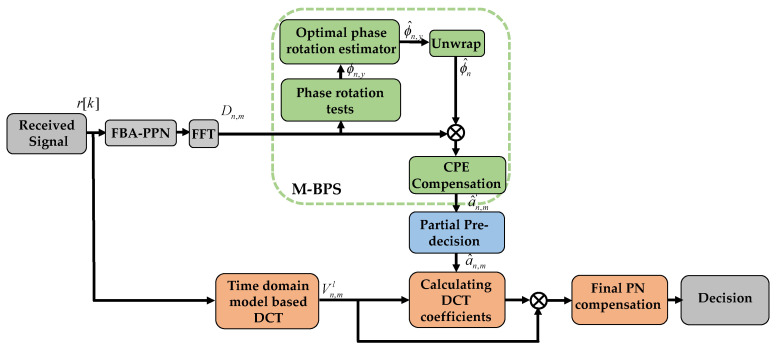
Block diagram of the proposed blind phase noise compensation scheme.

In this section, a blind PNC scheme is proposed to effectively compensate both CPE and ICI in CO-FBMC/OQAM systems. Figure 2 shows a block diagram of the proposed blind PNC scheme. In this method, a time-domain PNC model is firstly built by using the DCT approximation method, where each PN sample can be expanded as a linear combination of DCT coefficients and a group of DCT bases. To calculate these DCT coefficients in the time-domain model, it is necessary to pre-estimate the transmitted frequency-domain signals. As a result, the CPE is firstly compensated by using the M-BPS algorithm [28], and then the pre-decision process is performed on a part of the compensated signals to obtain the pre-estimated values of the transmitted signals.

After implementing PPN for filter bank analysis and FFT, the M-BPS method is firstly used to compensate the CPE. In the M-BPS method, the signal is firstly rotated by several test phases. The values of test phases are defined as $\phi_{n,y}=(y/Y)\cdot \pi -\pi /2$, where *y* = 1, 2, …, *Y*, and *Y* is the total number of test phases. The rotated version of $D_{n,m}$ can be given by

(10) $${\hat{D}}_{n,m,y}=D_{n,m}\cdot e^{-j\phi_{n,y}}$$

The estimated value of the CPE is obtained by minimizing the sum of distances between the hard decision of the received samples after test phase compensation and their projections on the real axis,

(11) ϕ^n,y=argminϕn,y∑m=0M−1|Re(D^n,m,y)−DD(Re(D^n,m,y))|

where Re(⋅) denotes the real projection operator, and DD(⋅) is the direct pre-decision operator. Subsequently, the conventional unwrap operation is performed to partly solve the problem of phase ambiguity [42]. Then, the received PAM symbol is obtained as follows:

(12) a^n,m′=Re(D^n,m)=Re(Dn,m⋅e−jϕ^n)

**Figure 3:**
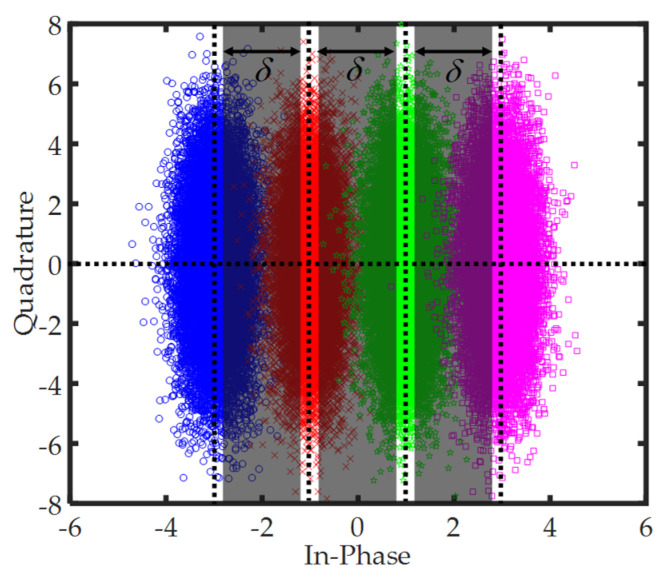
Taking the 16-OQAM as an example, the signals with high decision error probability in three shadow regions (${\hat{a}}_{n,m}^{\prime }\in [-2-\delta /2,-2+\delta /2]\cup [-\delta /2,\delta /2]\cup [2-\delta /2,2+\delta /2]$) not being used to perform the pre-decision.

Next, the estimated values ${\hat{a}}_{n,m}$ of the transmitted signal are recovered via the pre-decisions upon the signal ${\hat{a}}_{n,m}^{\prime }$. Since the performance of the proposed method strongly depends on the pre-decisions, the negative influence of the decision errors should be considered. Here, an effective approach is taken to reduce the decision errors. As shown in Figure 3, taking the 16-OQAM as an example, the constellation points are classified into 4 classes. When the constellation point ${\hat{D}}_{n,m}$ falls in the border regions between two neighbouring classes, the decision errors often occur. Then, three shadow border regions are regarded as the high decision error probability regions, where the parameter δ is defined as is the width of a shadow rectangle, and it decides the selected range of the high decision error probability regions. Therefore, if ${\hat{D}}_{n,m}$ falls in these high decision error probability regions, these signals are thrown away and not used to perform pre-decisions; while ${\hat{D}}_{n,m}$ does not fall in the shadow regions, a hard pre-decision is made normally. However, if excessive signals are removed from the pre-decision process, this can lead to the performance degradation of the proposed method. Thus, the pre-decision parameter δ needs to be optimized to achieve good performance. 

Secondly, a time-domain PNC model is built to estimate the PN with the aid of DCT. The compensated signal ${\hat{r}}_{n}[i]$ can be obtained by multiplying the received signal *r_n_*[*i*] by the complex conjugate of the estimate of PN,

(13) $${\hat{r}}_{n}[i]=r_{n}[i]e^{-j{\hat{\varphi }}_{n}[i]}$$

where *φ_n_*[*i*] denotes the *i*th time-domain PN sample in the *n*th CO-FBMC/OQAM block. The high frequency components in the PN can be neglected, and the complex conjugate of PN can be expressed as the linear combination of a group of DCT basis and DCT coefficients [37],

(14) $$\Phi_{n}\approx \tau C_{n}$$

where $\Phi_{n}={\left[e^{-j{\hat{\varphi }}_{n}[0]},e^{-j{\hat{\varphi }}_{n}[2]},\ldots ,e^{-j{\hat{\varphi }}_{n}[KM-1]}\right]}^{T}$ and $C_{n}={\left[C_{n}(0),C_{n}(1),\ldots ,C_{n}(L-1)\right]}^{T}$ are *L* × 1 unknown DCT coefficient vector. Here, ${\left[\cdot \right]}^{T}$ is the transpose operator, and *L* refers to the length of DCT coefficients. The *L_g_* × *L* matrix **τ** of DCT basis is given by

(15) $$\tau =\left[\begin{array}{ccccc} \frac{\sqrt{2}}{2} & \cos\left(\frac{\pi (1+0.5)}{KM}\right) & \cos\left(\frac{2\pi (1+0.5)}{KM}\right) & \cdots & \cos\left(\frac{\left(L-1)\pi (1+0.5\right)}{KM}\right)\\ \frac{\sqrt{2}}{2} & \cos\left(\frac{\pi (2+0.5)}{KM}\right) & \cos\left(\frac{2\pi (2+0.5)}{KM}\right) & \cdots & \cos\left(\frac{\left(L-1)\pi (2+0.5\right)}{KM}\right)\\ \frac{\sqrt{2}}{2} & \cos\left(\frac{\pi (3+0.5)}{KM}\right) & \cos\left(\frac{2\pi (3+0.5)}{KM}\right) & \cdots & \cos\left(\frac{\left(L-1)\pi (3+0.5\right)}{KM}\right)\\ \vdots & \vdots & \vdots & \ddots & \vdots \\ \frac{\sqrt{2}}{2} & \cos\left(\frac{\pi (KM+0.5)}{KM}\right) & \cos\left(\frac{2\pi (KM+0.5)}{KM}\right) & \cdots & \cos\left(\frac{\left(L-1)\pi (KM+0.5\right)}{KM}\right) \end{array}\right]$$

substituting Equation (14) into Equation (13), the compensated temporal signal ${\hat{r}}_{n}[i]$ can be rewritten as,

(16) $${\hat{r}}_{n}[i]=\sum_{l=0}^{L-1}r_{n}[i]\tau_{i,l}C_{n}(l)$$

hence, the frequency domain compenstated symbol after analysis processing can be expressed as,

(17) $$\begin{array}{cc} {\hat{R}}_{n,m} & =\sum_{i=0}^{L_{g}-1}{\hat{r}}_{n}[i]g[i-n\frac{M}{2}]\cdot e^{-j\frac{2\pi }{M}mi}e^{-j\frac{\pi (m+n)}{2}}\\ & =\sum_{i=0}^{L_{g}-1}e^{-j{\hat{\varphi }}_{n}[i]}r_{n}[i]g[i-n\frac{M}{2}]\cdot e^{-j\frac{2\pi }{M}mi}e^{-j\frac{\pi (m+n)}{2}}\\ & =\sum_{i=0}^{L_{g}-1}e^{-j{\hat{\varphi }}_{n}[i]}\left(e^{j\varphi_{n}[i]}s_{n}[i]+w_{n}[i]\right)g[i-n\frac{M}{2}]\cdot e^{-j\frac{2\pi }{M}mi}e^{-j\frac{\pi (m+n)}{2}}\\ & \approx \underset{A_{n,m}}{\underset{︸}{\sum_{i=0}^{L_{g}-1}s_{n}[i]g[i-n\frac{M}{2}]\cdot e^{-j\frac{2\pi }{M}mi}e^{-j\frac{\pi (m+n)}{2}}}}+\xi_{n,m} \end{array}$$

where *A_n,m_* is the received symbol when the PN is perfectly compensated — i.e., the transmitted PAM symbol *a_n,m_* can be recovered by taking real part of *A_n,m_* — and $\xi_{n,m}$ is the noise term. By replacing ${\hat{r}}_{n}[i]$ from Equation (16) into Equation (17), the symbol ${\hat{R}}_{n,m}$ is rewritten into

(18) $$\begin{array}{cc} {\hat{R}}_{n,m} & =\sum_{i=0}^{L_{g}-1}e^{-j{\hat{\varphi }}_{n}[i]}r_{n}[i]g[i-n\frac{M}{2}]\cdot e^{-j\frac{2\pi }{M}mi}e^{-j\frac{\pi (m+n)}{2}}\\ & =\sum_{i=0}^{L_{g}-1}\sum_{l=0}^{L-1}C_{n}(l)\tau_{i,l}r_{n}[i]g[i-n\frac{M}{2}]\cdot e^{-j\frac{2\pi }{M}mi}e^{-j\frac{\pi (m+n)}{2}}\\ & =\sum_{l=0}^{L-1}C_{n}(l)\sum_{i=0}^{L_{g}-1}r_{n}[i]g[i-n\frac{M}{2}]\tau_{i,l}e^{-j\frac{2\pi }{M}mi}e^{-j\frac{\pi (m+n)}{2}}\\ & =\sum_{l=0}^{L-1}C_{n}(l)V_{n,m}^{l} \end{array}$$

where the symbol $V_{n,m}^{l}$ satisfies

(19) $$V_{n,m}^{l}=\sum_{i=0}^{L_{g}-1}r_{n}[i]g[i-n\frac{M}{2}]\tau_{i,l}e^{-j\frac{2\pi }{M}mi}e^{-j\frac{\pi (m+n)}{2}}$$

By combining Equation (17) and Equation (18), the received symbol *A_n,m_* can be expressed as

(20) $$A_{n,m}\approx \sum_{l=0}^{L-1}C_{n}(l)V_{n,m}^{l}-\xi_{n,m}$$

While ignoring the noise term $\xi_{n,m}$, the corresponding estimated vector ${\hat{A}}_{n}$ can be approximated as,

(21) $${\hat{A}}_{n}=V_{n}C_{n}$$

where ${\hat{A}}_{n}={\left[{\hat{A}}_{n,0},{\hat{A}}_{n,1},\ldots ,{\hat{A}}_{n,M-1}\right]}^{T}$ and $V_{n}=[V_{n}^{0},V_{n}^{1},\ldots ,V_{n}^{L-1}]$ (m∈[0,M−1]). ${\hat{A}}_{n,m}$ is the estimated value of *A_n,m_*, and the *M* × 1 vector $V_{n}^{l}={\left[V_{n,0}^{l},V_{n,1}^{l},\ldots ,V_{n,M-1}^{l}\right]}^{T}$ is a column of the matrix $V_{n}$. 

Taking the real part of Equation (21) in the left and right sides, the equation is changed into

(22) $${\hat{a}}_{n}=P_{n}Q_{n}$$

where ${\hat{a}}_{n}=[{\hat{a}}_{n,0},{\hat{a}}_{n,1},\ldots ,{\hat{a}}_{n,M-1}]$, Pn=[Re(Vn)−Im(Vn)], Qn=[Re(CnT) Im(CnT)]T. ${\hat{a}}_{n,m}$ is the estimated value of the transmitted PAM symbol, and Im(⋅) denotes the imaginary projection operator. As above-mentioned, in order to reduce the decision errors, only partially estimated values of the transmitted signal can be obtained by making a pre-decision on the the constellation points in the low decision error probability regions. When the optimized parameter δ is selected by balancing performance and complexity of the proposed algorithm, a $Z_{n}\times 1$ vector is picked out to perform the pre-decision, where *Z_n_* is the total number of the pre-estimated transmitted signals in the *n*th FBMC/OQAM block. The pre-estimated transmitted signal vector can be given by $S_{n}{\hat{a}}_{n}$, where the permutation matrix $S_{n}={\left[\lambda_{t_{1}},\lambda_{t_{2}},\ldots ,\lambda_{t_{Z_{n}}}\right]}^{T}$ is a *Z_n_* × *M* matrix, *t_z_* (*z* = 1, 2, …, *Z_n_*) represents the subcarrier index of *z*th pre-estimated transmitted signal, and $\lambda_{t_{z}}$ is a *M* × 1 vector ${\left[\underset{t_{z}-1}{\underset{︸}{0,\ldots ,0}},1,\underset{M-t_{z}}{\underset{︸}{0,\ldots ,0}}\right]}^{T}$. Subsequently, Equation (22) can be rewritten as

(23) $$S_{n}{\hat{a}}_{n}=S_{n}P_{n}Q_{n}$$

Finally, the least square (LS) solution of the unknown DCT coefficient vector can be easily found as follows:

(24) $$Q_{n}={\left({\left(S_{n}P_{n}\right)}^{T}(S_{n}P_{n})\right)}^{-1}{\left(S_{n}P_{n}\right)}^{T}(S_{n}{\hat{a}}_{n})$$

Once the DCT coefficient vector ***Q****_n_* is obtained, the PN, including the CPE and ICI, can be compensated via Equation (22).

## 4. Modified Algorithm Based on Overlapped Symbol Structure

**Figure 4:**
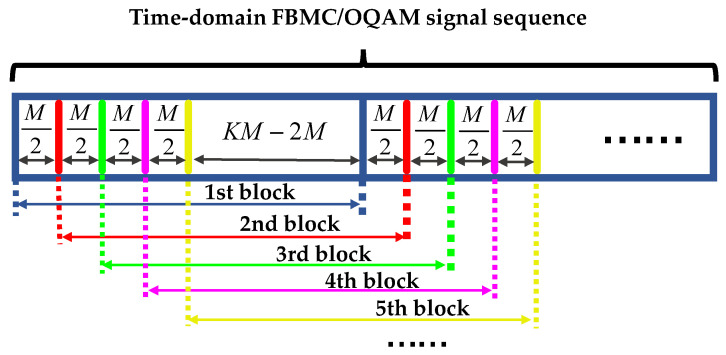
Time-domain FBMC/OQAM transmitted blocks with overlapped structure.

Different from OFDM symbols, neighboring temporal FBMC/OQAM blocks are overlapped with each other. Figure 4 shows the time-domain FBMC/OQAM transmitted blocks with overlapped structure. The *i*th sample of *n*th received FBMC/OQAM block can be given by

(25) $$r_{n}[i]=r[i+(n-1)\frac{M}{2}],\;i\in [1,KM]$$

Then, the last *KM-M/2* samples of *n*th FBMC/OQAM block are the same as the first *KM-M/2* samples of (*n +* 1)th FBMC/OQAM block—i.e., $r_{n}[j+M/2]=r_{n+1}[j]$, j∈[1,KM−M/2]. As a consequence, the PN sample also satisfies that $\varphi_{n}[j+M/2]=\varphi_{n+1}[j]$, where $\varphi_{n+1}[j]$ is the *j*th sample of the (*n* + 1)th PN vector $\varphi_{n+1}$. Let $\tau_{pre}$ and $\tau_{last}$ be the first and the last *KM-M/2* rows of DCT basis τ, repectively, and we can obtain that $\tau_{last}C_{n}=\tau_{pre}C_{n+1}$. Using LS estimation, the DCT coefficient vector ***C****_n_* for *n*th CO-FBMC/OQAM block can be obtained as follows,

(26) $$C_{n}={\left(\tau_{last}^{T}\tau_{last}\right)}^{-1}\tau_{last}^{T}\tau_{pre}C_{n+1}$$

If the first DCT coefficient vector ***C***_1_ is known, the other DCT coefficient vectors can be calculated according to the recursive expression in Equation (26). It is obviously unreasonable in such a way to calculate the other DCT coefficient vectors, because the updating between the PN vectors of $\varphi_{n}$ and $\varphi_{n+1}$ is not completely neglected. Moreover, two truncated matrices of $\tau_{pre}$ and $\tau_{last}$ have very small singular values owing to the circularity of the DCT basis matrix τ. This leads to the significant degradation of the accuracy in the LS estimation. Therefore, an approximate expression between the DCT coefficient vectors of ***C****_n_* and ***C****_n_*_+1_ is derived to achieve high estimate accuracy. 

Although the DCT basis matrix is not an orthogonal matrix in the proposed algorithm, the following condition is still satisfied:

(27) $$\tau^{T}\tau =bI$$

where *b* is a constant, and **I** is a *L* × *L* identity matrix. When the difference between the first and the last *M*/2 samples of $\varphi_{n}$ and $\varphi_{n+1}$ are ignored, $\varphi_{n+1}$ can be approximated as the *M*/2 circular shift of $\varphi_{n}$. Hence, the terms satisfy that

(28) $$\{\begin{array}{c} \tau C_{n}=\varphi_{n}\approx \mu_{-1}\varphi_{n+1}=\mu_{-1}\tau C_{n+1}\\ \tau C_{n+1}=\varphi_{n+1}\approx \mu_{+1}\varphi_{n}=\mu_{+1}\tau C_{n} \end{array}$$

where $\mu_{-1}$ and $\mu_{+1}$ are two *L_g_* × *L_g_* circular shift matrices,

(29) $$\left\{ \begin{array}{c} \mu_{-1}=\left[\begin{array}{cc} 0_{\frac{M}{2}\times (L_{g}-\frac{M}{2})} & I_{\frac{M}{2}}\\ I_{L_{g}-\frac{M}{2}} & 0_{(L_{g}-\frac{M}{2})\times \frac{M}{2}} \end{array}\right]\\ \mu_{+1}=\left[\begin{array}{cc} 0_{(L_{g}-\frac{M}{2})\times \frac{M}{2}} & I_{L_{g}-\frac{M}{2}}\\ I_{\frac{M}{2}} & 0_{\frac{M}{2}\times (L_{g}-\frac{M}{2})} \end{array}\right] \end{array}\right.$$

here, **0***_m_*_ × *n*_ denotes a *m* × *n* zero matrix, and **I***_n_* is a *n* × *n* identity matrix. In combination with Equation (27), Equation (28) can be further expressed as

(30) $$\{\begin{array}{c} C_{n}=\gamma_{-1}C_{n+1}\\ C_{n+1}=\gamma_{+1}C_{n} \end{array}$$

(31) $$\left\{ \begin{array}{l} \gamma_{-1}=\frac{1}{b}\tau^{T}\mu_{-1}\tau \\ \gamma_{+1}=\frac{1}{b}\tau^{T}\mu_{+1}\tau \end{array}\right.$$

Assuming that three neighboring FBMC/OQAM blocks participate in the estimation process, Equation (21) can be expanded as

(32) $$\left[\begin{array}{c} {\hat{A}}_{n-1}\\ {\hat{A}}_{n}\\ {\hat{A}}_{n+1} \end{array}\right]=\left[\begin{array}{c} V_{n-1}\gamma_{-1}\\ V_{n}\\ V_{n+1}\gamma_{+1} \end{array}\right]C_{n}$$

The matrices of ${\bar{a}}_{n}$ and ${\bar{P}}_{n}$ are separately defined as ${\bar{a}}_{n}={\left[{\hat{a}}_{n-1}^{T},{\hat{a}}_{n}^{T},{\hat{a}}_{n+1}^{T}\right]}^{T}$ and

(33) P¯n=[Re(Vn−1γ−1)−Im(Vn−1γ−1)Re(Vn)−Im(Vn)Re(Vn+1γ+1)−Im(Vn+1γ+1)].

Similar to Equations (22)–(24), the LS solution of the unknown DCT coefficient vector can be obtained from the following expression,

(34) $$Q_{n}={\left({\left({\bar{S}}_{n}{\bar{P}}_{n}\right)}^{T}({\bar{S}}_{n}{\bar{P}}_{n})\right)}^{-1}{\left({\bar{S}}_{n}{\bar{P}}_{n}\right)}^{T}({\bar{S}}_{n}{\bar{a}}_{n})$$

where ${\bar{S}}_{n}={\left[S_{n-1}^{T},S_{n}^{T},S_{n+1}^{T}\right]}^{T}$. 

In the modified proposed algorithm, we throw away the first *M*/2 samples of the (*n* − 1)th PN vector $\varphi_{n-1}$ and the last *M*/2 samples of the (*n* + 1)th PN vector $\varphi_{n+1}$; in spite of this, the relatively small power is on both ends of impulse response of the prototype filter so that the approximation affects rarely its performance, as the vector ***C****_n_* is obtained in such a way, and the vectors of ***C***_*n* − 1_ and ***C***_*n* + 1_ are easily calculated using the recurrence relations in Equations (30)–(31). Therefore, only one inverse operation in Equation (34) is required for every three neighboring FBMC/OQAM blocks, and the complexity can be decreased by the simplified operation. The detailed complexity analysis will be described in the next section. The acronyms used in this article are summarized in the following Table 1. 

## 5. Simulation Results and Discussion

A 30 GBaud BTB CO-FBMC/OQAM transmission system is simulated to evaluate the performance of the proposed blind PNC scheme, which is built by Optisystem 17.0 and MATLAB. Firstly, a pseudo-random bit sequence with a word length of 2^17^ is mapped into m-QAM and the symbol rate is set to 30 GBaud/s. All the complex-valued QAM symbols are converted into the real-valued PAM symbols through QAM to PAM module. The base-band time-domain FBMC/OQAM symbols are generated after all PAM data sequences through IFFT and FBS-PPN. The pulse length of the square root raised cosine filter is chosen as *L_g_* = 4*M*, and its roll-off coefficent α=1. 

Then, the base-band FBMC/OQAM signals are modulated onto an optical carrier at 1550 nm using a Mach–Zehnder modulator with a pair of independently controllable branches. At the receiver, the optical signal is coherently detected in a 90° optical hybrid, and the received analog signals are converted into digital signals by analog-to-digital converters with two samples per symbol. Subsequently, the obtained digital symbols are fed into the offline DSP module. With time synchronization being perfectly implemented, all the time-domain symbols are converted to the frequency domain by the PPN for filter bank analysis and FFT. In this paper, perfect channel equalization is considered, and specifically, we suppose that the channel response is flat and time-invariant on each carrier [27]. Next, the blind discrete-cosine-transform-based phase noise compensation (BD-PNC) is implemented, including CPE and ICI compensation. In the related M-BPS algorithm, the total numbers of test phases for 4/16/64-QAM are chosen as 16/32/64, respectively [28]. The original binary bits can be recovered after m-OQAM and m-QAM de-mapping, and the bit error rate (BER) is estimated by comparing them with the transmitted bits.

**Figure 5:**
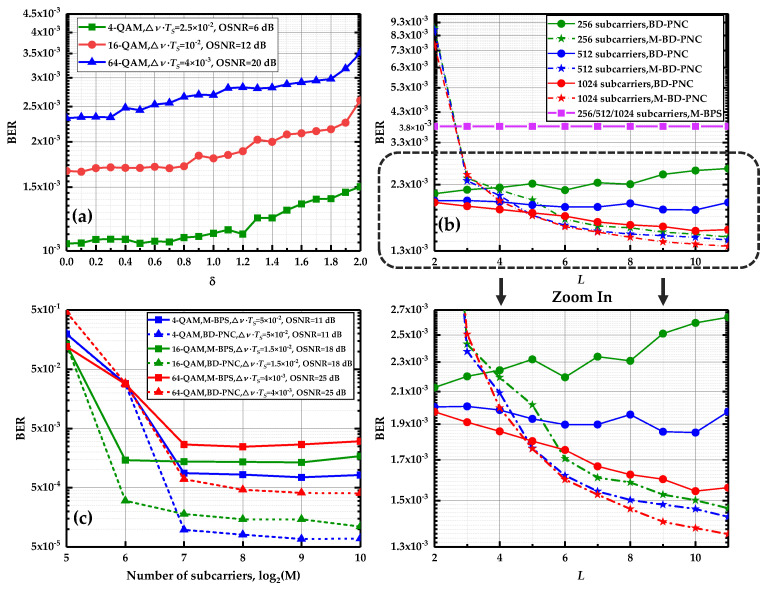
(**a**) BER versus the width of shadow rectangle in a high decision error probability region, *δ*. (**b**) BER versus the length of DCT coefficient, *L*. (**c**) BER versus the number of subcarriers, *M*.

The effects of two parameters of *δ* and *L* on the performance of the algorithm are investigated in Figure 5a,b. *δ* is the width of shadow rectangle in a high decision error probability region, which determines how many signals after CPE compensation join the pre-decision process. As mentioned in Section 3, *δ* = 0 means that all the data are used to perform the pre-decision while *δ* = 2 implies that only the leftmost and rightmost data in the I-Q plane participate in the process in Figure 3. The decision error increases with the participation of more data after the CPE compensation. In Figure 5a, *δ* is optimized at the length of DCT basis *L* = 2 for the CO-FBMC 4/16/64-QAM BTB system with 512 subcarriers. When the value of *δ* is changed from 0 to 1.8, it can be seen that the BER performance does not degrade significantly due to the reduction in decision error probability. Moreover, with the increase in the value of *δ*, the participation of a low number of data benefits greatly to complexity reduction in the proposed algorithm. Hence, *δ* = 1.8 is adopted in the remainder of this paper. *L* denotes the length of the DCT basis. Generally, under a large value of *L*, we can obtain a more accurate approximation of the PN by the DCT in Equation (14). Figure 5b displays the BER performance as a function of *L* at the linewidth and symbol duration product $∆\nu \cdot T_{S}=10^{-2}$ with 12 dB optical signal-to-noise ratio (OSNR) and 256/512/1024 subcarriers when using the proposed BD-PNC algorithm and its modified version. The modified algorithm is developed by considering the overlapped symbol structure, which is called the M-BD-PNC scheme. For the BD-PNC, with the increase in *L*, the BER performance makes a small improvement, and is even deteriorated for the 256/512 subcarrier FBMC/OQAM systems. While using the M-BD-PNC method, there is a significant improvement for its BER performance as the value of *L* is increased. The PN estimation precision depends on the accuracy of the DCT coefficients estimated by the LS method. In Equation (24) or Equation (34), if the matrix $S_{n}P_{n}$ or ${\bar{S}}_{n}{\bar{P}}_{n}$ does not meet the column full-rank condition, its LS solution could produce a large error. With the increase in the number of subcarriers, more rows in the matrix allow it to meet the column full-rank condition more easily. Hence, when *L* is increased, the proposed BD-PNC algorithm achieves better performance under a large number of subcarriers; its performance becomes worse in the system with a low number of subcarriers, while in the M-BD-PNC method, ${\bar{P}}_{n}$ is the extended version of $P_{n}$ so that it is easier for the matrix ${\bar{S}}_{n}{\bar{P}}_{n}$ to satisfy a column full-rank term. In this case, the PN estimation precision is almost not negatively influenced by the LS method, and a large value of *L* contributes to the improvement of the PN estimation precision. Therefore, for the M-BD-PNC, the BER performance exhibits a better result at a large *L*. The optimized values of *L* in the BD-PNC and M-BD-PNC are selected as 2 and 10, respectively.

The CO-FBMC/OQAM system with a large number of subcarriers is robust against fiber CD [12]. However, the PN becomes strong with the increase in the number of subcarriers. It is always a challenge for the existing PNC algorithm to achieve good performance under a large number of subcarriers [18,25,26,27,28,29]. So the performance of the proposed algorithm is evaluated in the BTB system with a large number of subcarriers. In Figure 5c, the influence of the number of subcarriers on the performance of the method is investigated at *L* = 2 and *δ* = 1.8 using M-BPS and BD-PNC for the CO-FBMC 4/16/64 QAM systems with several different values of $∆\nu \cdot T_{S}$ and OSNR. When the number of subcarriers becomes larger, on one hand, the effect of AWGN is better averaged so that their performance is improved rapidly; on the other hand, the FBMC/OQAM symbol duration increases, and the PN has a larger variance and becomes stronger. As a result, it can be seen that the BER using M-BPS starts to deteriorate at *M* = 1024. Note that the performance using BD-PNC remains almost unchanged when the number of subcarriers is increased from 128 to 1024 for 4/16/64-QAM modulation order. The simulation results prove that its performance is not sensitive to the number of subcarriers in the CO-FBMC/OQAM systems. Under the large number of subcarriers, the proposed algorithm is far superior to the M-BPS. For the 4-QAM modulation at *M* = 1024 with $∆\nu \cdot T_{S}=5\times 10^{-2}$ and 11 dB OSNR, the BER performance of BD-PNC is improved by more than one order of magnitude compared to the M-BPS.

**Figure 6:**
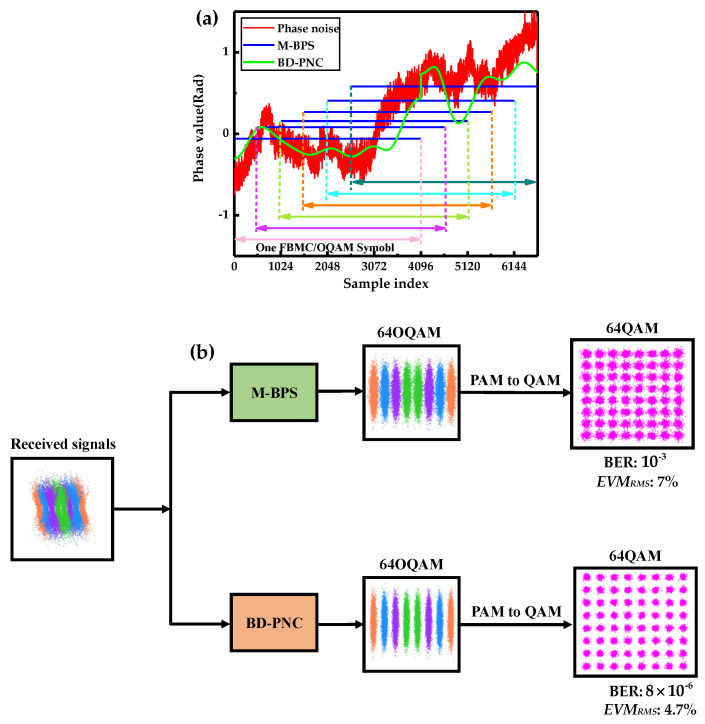
(**a**) One realization of the real phase noise and its estimations after the M-BPS and BD-PNC methods at $∆\nu \cdot T_{S}=1.5\times 10^{-2}$ and OSNR = 20 dB for 30 Gbaud CO-FBMC 16-QAM BTB transmission systems with 1024 subcarriers. (**b**) Illustration of constellations before and after PNC employing M-BPS and BD-PNC at $∆\nu \cdot T_{S}=5\times 10^{-3}$ and OSNR = 27 dB for 30 Gbaud CO-FBMC 64-QAM BTB systems with 1024 subcarriers.

Figure 6a depicts one realization of the real PN and its estimation within six consecutive FBMC/OQAM blocks at the linewidth and symbol duration product $∆\nu \cdot T_{S}=1.5\times 10^{-2}$ and an OSNR of 20 dB for 1024 sub-carrier 16-QAM CO-FBMC systems using the BD-PNC and M-BPS. For these time-domain overlapped symbols, the CPE estimated by the M-BPS is only a rough estimate of the PN, which is far from the real PN. The proposed method shows a better phase tracking capability due to the ICI compensation in time-domain. Figure 6b shows the illustration of ICI influence and the necessity of PN estimation in time domain for CO-FBMC/OQAM systems. The constellation diagrams before and after PNC are separately displayed using M-BPS and BD-PNC with an OSNR of 27 dB and combined laser linewidth of 150 kHz. The PN in time domain, including CPE and ICI, is compensated by using BD-PNC, while only CPE is compensated by using M-BPS. It is clear that the PN in time domain is compensated by the proposed BD-PNC method resulting in an improvement of about 125 times in terms of BER over the M-BPS. Moreover, the BD-PNC achieves a root-mean-square error vector magnitude (*EVM_RMS_*) of 4.7%, which is much lower than the *EVM_RMS_* of 7% by the M-BPS.

**Figure 7:**
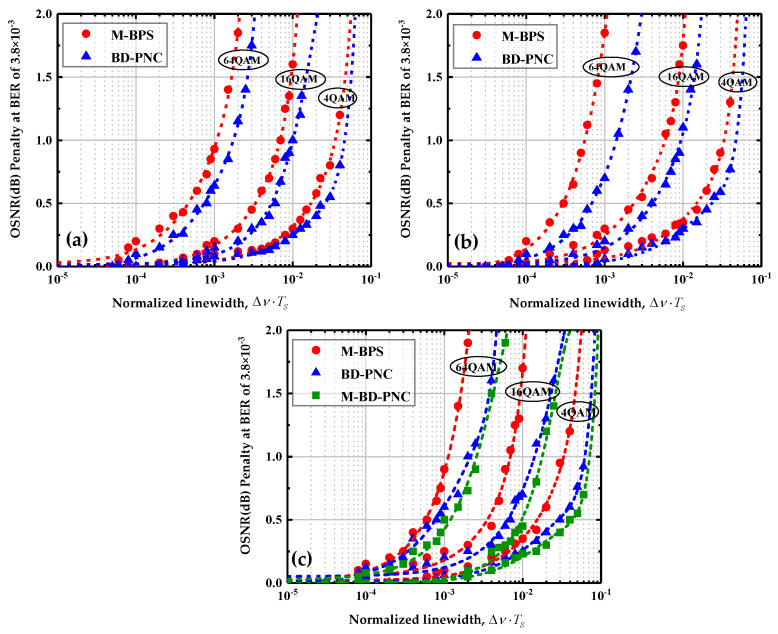
OSNR penalty versus $∆\nu \cdot T_{S}$ at a BER of $3.8\times 10^{-3}$ using M-BPS, the proposed schemes for CO-FBMC 4/16/64-QAM systems with 256 subcarriers (**a**), 512 subcarriers (**b**), and 1024 subcarriers (**c**).

Supposing that the pre-forward error correction BER should be lower than $3.8\times 10^{-3}$ for hard-decision forward error correction (HD-FEC) [43], the performance of these PNC algorithms is investigated at the BER target limit. Figure 7a–c show the OSNR penalty as a function of the linewidth and symbol duration product $∆\nu \cdot T_{S}$ using M-BPS and the proposed algorithms for CO-FBMC/OQAM BTB transmission system with 256, 512, and 1024 subcarriers, respectively. The optimal numbers of test phases in the related M-BPS scheme are chosen as 16/32/64 for 4/16/64-QAM modulation order, respectively [28]. When the number of subcarriers is 256 or 512, the length *L* of DCT basis is set to 2. The length of DCT basis *L* is selected as 10 with total number of subcarriers *M* = 1024. The parameter *δ* is set to 1.8 in this paper. The OSNR penalty is defined as the difference between the required OSNR for a BER of $3.8\times 10^{-3}$ at a current laser linewidth and zero laser linewidth in CO-FBMC/OQAM systems, where the PNC algorithm is used in the former, and none of the PNC methods is applied in the PN free system in the latter. When no PN exists in the system, the reference OSNRs for 4/16/64-QAM 30 Gbaud signal at the HD-FEC threshold are ∼4.4, ∼10, and∼16.8 dB, respectively. For comparison, an acceptable maximum of 1 dB OSNR penalty is regarded as a threshold value [27].

On the whole, the proposed algorithm provides significantly better performance than M-BPS. In Figure 7a, at 1 dB OSNR penalty threshold, with the number of subcarriers *M* = 256, the M-BPS achieves a $∆\nu \cdot T_{S}$ tolerance of $3.5\times 10^{-2}$, $7\times 10^{-3}$, and $10^{-3}$ for 4/16/64-QAM 30 Gbaud signal, respectively. While using the proposed BD-PNC method, under 1 dB OSNR penalty constraint, the maximum $∆\nu \cdot T_{S}$ that can be achieved for 4/16/64-QAM are $5\times 10^{-2}$, $10^{-2}$, and $1.8\times 10^{-3}$, respectively. With the increase in the QAM modulation order, the difference between the performance of the two methods is more remarkable. At higher order modulation format, the ICI effect becomes stronger, and the BD-PNC scheme achieves more significant results than M-BPS. In Figure 7b, at *M* = 512, M-BPS and BD-PNC achieve a $∆\nu \cdot T_{S}$ tolerance of $5.6\times 10^{-4}$ and $1.4\times 10^{-3}$ for 64-QAM 30 Gbaud signal, respectively.

The studies focus on the investigation of the proposed BD-PNC algorithm’s performance with the number of subcarriers *M* = 1024. As a comparison, the performance of the M-BD-PNC algorithm is also evaluated in the 30 GBaud BTB CO-FBMC/OQAM systems with 1024 subcarriers. In Figure 7c, the M-BD-PNC achieves a better $∆\nu \cdot T_{S}$ tolerance than the BD-PNC. This is because the column full-rank condition is easily satisfied in the M-BD-PNC scheme, and its performance benefits from the enhanced estimation accuracy of DCT coefficients by the LS method. Moreover, the M-BD-PNC has a lower CC than the corresponding BD-PNC algorithm, and a detailed analysis of their CC will be described at the end of the section. With a large number of subcarriers, the ICI components in PN have a huge negative impact on the performance. With the ICI effect mitigated, the proposed algorithms offer a significantly better performance compared to the M-BPS under the same QAM modulation order. In particular, the BD-PNC and M-BD-PNC achieve tolerated linewidth and symbol duration products of $2.2\times 10^{-2}$ and $2.6\times 10^{-3}$ for 64-QAM 30 Gbaud signal, while the M-BPS can only achieve a $∆\nu \cdot T_{S}$ tolerance of $10^{-3}$.

Finally, we compare the CC of M-BPS, the proposed BD-PNC, and M-BD-PNC algorithms in terms of the number of real multiplications in one FBMC/OQAM block. Since the M-BD-PNC method involves several neighboring FBMC/OQAM blocks, its CC is evaluated as the average CC per block. As shown in Table 2, the CC of the proposed BD-PNC and M-BD-PNC algorithms are compared. According to the block diagram in Figure 2, the CC of the proposed BD-PNC involves the CC of its four DSP steps, including M-BPS, time-domain PNC, calculation of DCT coefficients, and final compensation. The CC of the M-BPS method is given by *O*(2*MY*) [28]. The time-domain PNC model is described by Equation (19), and its CC is *O*(*LM*log_2_*M*+*LKM*). For the BD-PNC, the DCT coefficients are calculated in Equation (24), and its CC is given by *O*(8*L*^3^ + 8*Z_n_L*^2^ + 2*Z_n_L*), where *Z_n_* is the total number of pre-estimated transmitted signals in the *n*th FBMC/OQAM block, while in the M-BD-PNC, the DCT coefficients are obtained by Equations (30) and (34), and real multiplications needed in these equations are *O*(8*L*^3^/3 + 8*Z_n_L*^2^ + 2*ZnL* + 2*L*^2^/3). For BD-PNC and M-BD-PNC, the final PNC is completed by using Equation (22), and its CC is *O*(2*ML*). In order to compare the CC of these algorithms directly, several simulations are carried out to investigate the average value of *Z_n_* at a BER of $3.8\times 10^{-3}$. In this case, the average value of *Z_n_* is shown in Table 3. Consequently, the required real multiplication numbers per block are counted by type, as shown in Table 4. As a comparison, when all the data after CPE compensation participate in the pre-decision (*δ* = 0), the required real multiplication numbers of BD-PNC or M-BD-PNC is provided in parentheses. It is observed that the optimization of the parameter *δ* in the pre-decision process results in a remarkable decrease in complexity. Furthermore, it is clear that the CC of proposed BD-PNC or M-BD-PNC is not significantly higher than M-BPS, and their CC is less than four times higher than that of M-BPS for 1024 subcarriers 64-QAM 30 Gbaud signals. Nevertheless, the proposed algorithms have a great advantage over M-BPS in terms of the PNC performance. The CC of M-BD-PNC is slightly lower than that of BD-PNC, and its performance is even superior to the BD-PNC algorithm. Therefore, at *M* = 1024, the proposed M-BD-PNC should be the first choice.

## 6. Conclusions

In this paper, we have presented a blind time-domain PNC scheme for the CO-FBMC/OQAM transmission, where the PN sample is approximately expanded by DCT. In the proposed algorithms, the PN in time-domain can be compensated by obtaining their corresponding DCT coefficients. The pre-decision process is firstly performed over the selected signals after CPE compensation by M-BPS, and the decision results are used to calculate these DCT coefficients. This selective decision process reduces the CC largely. Considering the overlapped symbol structure, the modified version of the proposed algorithm is reported with its performance improvement and complexity decrease. Since the proposed algorithms are devoted to removing the ICI effect, they achieve significant performance improvement compared to M-BPS for a simulated 30 GBaud CO-FBMC/OQAM system, especially with a large number of subcarriers and high order QAM modulation format. The algorithm complexity analysis shows that its CC is slightly higher than M-BPS.

## Figures and Tables

**Table 1:** A summary of abbreviation.

Abbreviation	Full Name
CO-FBMC/OQAM	Coherent optical offset-quadrature amplitude modulation-based filter-bank multicarrier
MCM	multicarrier modulation
OFDM	orthogonal frequency division multiplexing
CO-OFDM	coherent optical OFDM
PN	phase noise
CPE	common phase error
ICI	inter-carrier interference
PNC	Phase noise compensation
IMI	intrinsic imaginary interference
SE	spectral efficiency
IFFT	inverse fast Fourier transform
FFT	fast Fourier transform
CC	computational complexity
BPS	blind phase search
M-BPS	modified BPS
BD-PNC	blind discrete-cosine-transform-based phase noise compensation
M-BD-PNC	modified BD-PNC
CD	chromatic dispersion
OBE	orthogonal-basis-expansion
DCT	discrete cosine transform
BTB	back to back
LS	least square
OSNR	optical signal-to-noise ratio
HD-FEC	hard-decision forward error correction
PPN	poly-phase network
DSP	digital signal processing
AWGN	additive Gaussian white noise
BER	bit error rate
*EVM_RMS_*	root-mean-square error vector magnitude

**Table 2:** The CC of the proposed BD-PNC and M-BD-PNC algorithms.

DSP Step	CC (BD-PNC)	CC (M-BD-PNC)
CPE pre-compensation (M-BPS)	*O*(2*MY*)	*O*(2*MY*)
Time-domain PNC model	*O*(*LM*log_2_*M* + *LKM*)	*O*(*LM*log_2_*M* + *LKM*)
Calculation of DCT coefficients	*O*(8*L*^3^ + 8*Z_n_L*^2^ + 2*Z_n_L*)	*O*(8*L*^3^/3 + 8*Z_n_L*^2^ + 2*Z_n_L* + 2*L*^2^/3)
Final compensation	*O*(2*ML*)	*O*(2*ML*)

Note: *M*: number of subcarriers. *Y*: number of test phases. *L*: length of DCT coefficient. *K*: overlap factor. *Z_n_*: total number of pre-estimated transmitted signals.

**Table 3:** The average value of *Z_n_*.

	*M* = 256	*M* = 512	*M* = 1024
4-QAM	140	280	570
16-QAM	80	160	320
64-QAM	40	90	180

**Table 4:** The required real multiplication numbers per block using several algorithms.

	M-BPS	BD-PNC	M-BD-PNC
4-QAM	8192|*M* = 256	20464 (24640)|*M* = 256	666740 (1039000)|*M* = 1024
	16384|*M* = 512	41888 (50240)|*M* = 512	
	32768|*M* = 1024	672008 (1044288)|*M* = 1024	
16-QAM	16384|*M* = 256	26496 (32832)|*M* = 256	494510 (1071800)|*M* = 1024
	32768|*M* = 512	53952 (66624)|*M* = 512	
	65536|*M* = 1024	499776 (1077056)|*M* = 1024	
64-QAM	32768|*M* = 256	41440 (49216)|*M* = 256	445245 (1137300)|*M* = 1024
	65536|*M* = 512	84200 (99392)|*M* = 512	
	131072|*M* = 1024	450512 (1142592)|*M* = 1024	
